# Adequately iodized salt is an important strategy to prevent iodine insufficiency in pregnant women living in Central Java, Indonesia

**DOI:** 10.1371/journal.pone.0242575

**Published:** 2020-11-19

**Authors:** Ina Kusrini, Jessica Farebrother, Donny Kristanto Mulyantoro

**Affiliations:** 1 Health Research and Development Magelang, Indonesia Ministry of Health, Magelang, Indonesia; 2 Department of Women and Children’s Health, King’s College London, London, United Kingdom; 3 Human Nutrition Laboratory, Institute of Food, Nutrition and Health, ETH Zürich, Zürich, Switzerland; Boston University School of Medicine, UNITED STATES

## Abstract

Iodine is an essential micronutrient for cognitive development and growth. Optimal intakes are critical during pregnancy. We report the iodine status and thyroid function of pregnant women living in areas previously affected by severe iodine deficiency and in longstanding iodine sufficient areas in Java, Indonesia. This cross-sectional study was conducted in Magelang, Java, from July to November 2015, in four sub-districts; two previously affected by severe iodine deficiency (area 1) and two that were iodine-sufficient (area 2). Iodine intake was estimated using median urinary iodine concentration in spot samples and mean urinary iodine excretion in 3 x 24-hour samples, thyroid hormones (thyroid-stimulating hormone and free thyroxine) were measured in venous blood samples, and iodine content of household salt samples was estimated by titration. We recruited a total of 244 pregnant women, 123 in area 1 and 121 in area 2. Urinary iodine results suggested adequate habitual iodine intakes in both areas (median urinary iodine concentration in area 1: 222 μg/l (interquartile range 189, 276 μg/l), area 2: 264 μg/l (interquartile range 172, 284 μg/l), however, the risk of inadequate intakes increased with advancing trimester (Odds Ratio = 2.59 (95% CI 1.19–5.67) and 3.85 (95% CI 1.64–9.02) at second and third trimesters, respectively). Estimated prevalence of thyroid function disorders was generally low. Salt was iodized to approximately 40 ppm and foods rich in native iodine did not contribute significantly to dietary intakes. Adequately iodized salt continues to prevent iodine insufficiency in pregnant women living in areas previously affected by severe iodine deficiency in Java, Indonesia. Monitoring and surveillance, particularly in vulnerable groups, should be emphasized to ensure iodine sufficiency prevails.

## Introduction

Iodine is an essential micronutrient, required for the synthesis of thyroid hormone. Pregnant women are more vulnerable to iodine deficiency than the general population and an optimal iodine intake during gestation can help to prevent the sequelae of iodine deficiency, known as the Iodine Deficiency Disorders (IDD). These include irreversible brain damage with neurological abnormalities such as cretinism if iodine deficiency during pregnancy is severe [1]. Mild gestational iodine deficiency may also affect cognitive development and growth, though studies to date are equivocal [2–4]. WHO/UNICEF/IGN recommends a daily iodine intake of 250 μg to maintain sufficiency during pregnancy [5, 6].

In 1980, Indonesia was classed as severely iodine-deficient based on the total goiter rate from surveys in school-aged children (SAC) [7, 8]. In addition to distribution of 100 mg oral iodized oil capsules, Universal Salt Iodization (USI) has been mandatory in Indonesia since 1994 [9].

National Health Surveys conducted in 2003, 2007, and 2013 revealed that the iodine status in SAC was optimal at the national level [10–12], and distribution of iodized oil ceased in 2009. However; the 2013 Indonesian Basic Health Research showed a USI coverage of 77%, with only 43% of samples being adequately iodized to the Indonesian national standard of 30 ppm [11]. Furthermore, the median urinary iodine concentration (MUIC) in pregnant women suggested a continued risk of deficiency, with MUIC indicating borderline sufficiency in both rural (151 μg/L) and urban (163 μg/L) areas and more than 24% of pregnant women were categorized with UIC <150 μg/L at national level [11, 13]. Moreover, it is well-known that pockets of risk of inadequate or excessive iodine intakes can exist at regional or local levels despite an optimal national picture. Disaggregated data from 2013 and 2014 in two sub-districts in Magelang, Central Java, a region previously endemic for iodine deficiency, suggested that iodine intakes in pregnant women were borderline sufficient [14, 15].

Given the 2013–2014 data, we hypothesized that pregnant women living in areas previously affected by severe iodine deficiency would have lower habitual iodine intakes than pregnant women living elsewhere in central Java. Therefore, the present study aimed to assess the iodine status and thyroid function of pregnant women living in areas previously affected by severe iodine deficiency. We also assess the consumption of iodized salt and iodine-containing foods, with specific consideration to the increased iodine requirement during pregnancy.

## Materials and methods

### Study design

This cross-sectional study was conducted in pregnant women living in four sub-districts of Magelang, Central Java between July and November 2015.

### Setting

#### Previously iodine deficient area, area 1

Sawangan and Bandongan are rural farming communities in mountainous areas that have a history of severe iodine deficiency [16], with total goiter rates (TGR) of over 70% and 50% recorded in the 1970’s, respectively [17]. Household iodized salt is the main source of iodine in these areas [18], and recent survey data suggests iodine sufficiency in SAC [13, 18].

#### Iodine sufficient area, area 2

Mungkid and Borobudur, are sub-urban areas with a dietary iodine intake from iodized salt in addition to other sources such as seafood. There is no history of IDDs in these communities with longstanding TGRs of less than 3% [19].

### Participants

The inclusion criteria for all subjects were: 1. confirmed pregnancy (gestational weeks 0–40); 2. age 16–44 years; 3. no exposure to iodized oil capsules in the last five years; 4. no reported chronic illnesses, or health state that could disturb the collection and/or measurement of the biomarkers collected in this study (UIC, TSH, fT4), as assessed by a physician. The first day of the last normal menstrual cycle was used to estimate the gestational week. Based on the trimester classification defined by the American College of Obstetricians and Gynecologists (ACOG) [20], women were allocated to trimesters: trimester 1: 0–13 weeks; trimester 2: 14–27 weeks; trimester 3: 28–40 weeks. Finally, we assessed the general nutritional status of each woman using the mid-upper arm circumference (MUAC). Pregnant women with a MUAC <23.5 were defined as having chronic energy malnutrition, and were referred for medical attention, however, were allowed to participate in the study.

We identified eligible women in a two-stage cluster sampling method [21], using the total number of villages, 29 in area 1 and 36 in area 2, as the primary sampling unit for each cluster. For the sampling frame, all eligible women were stratified by trimester in each cluster. Simple random sampling was used to choose a subject from the sampling frame to obtain the representative sample for each stratum. A cross-sectional study with a population-based sampling frame, based on the 2013 National Health Survey and results from a previous iodine study in pregnant women [10], using the Lemeshow formula for stratification surveys, we estimated a minimum sample size of 122 women per area would be required to estimate the prevalence of iodine deficiency in this study with an 80% power and 10% error [22]. Based on proportional allocation, 36 women were taken from trimester 1; 36 from trimester 2; and 18 from trimester 3 to obtain the minimum sample from each area.

The study and all procedures were approved by the Indonesian Health Research and Development Ethics Committee. All participants provided written informed consent before participation.

#### Study procedures and laboratory measurements

*Urine samples*. To assess iodine status in participating women, we used two types of urine collection: urinary iodine concentration (UIC) in spot urine samples, and urinary iodine excretion (UIE) in three 24-hour urine collections, taken on a Sunday, Tuesday, and Thursday. UIE samples were verified by volume and mixed by inversion before being aliquoted. Aliquots were prepared from UIC and UIE samples in triplicate and frozen a -18°C before analysis. Urinary iodine analysis based on the ammonium persulfate digestion Sandel-Kolthoff reaction was performed. The Indonesian National Research and Development Laboratory of the Indonesian National Health Research and Development Unit in Magelang (Indonesia, BP2GAKI Magelang), was successfully involved in the Urinary Iodine Quality Ensurement (EQUIP) program (US Center for Disease Control and Prevention, Atlanta). Both the UIC from spot samples and UIE from 24 h samples were used to estimate habitual iodine intake.

*Blood samples*. We measured thyroid function using thyroid-stimulating hormone (TSH) and free thyroxine (fT4) in venous blood samples. Participants were asked to provide a 5 ml blood sample, which was obtained by venipuncture by a trained laboratory technician. Serum was separated by centrifugation, and used to measure TSH and fT4 by Enzyme-Linked Immunoassay (ELISA), using the Human Reagent kit (Human Gesselschaft fur Biochemica and Diagnostica mbH, Max–Planck-Ring21 65205 Wiesbaden, Germany) at the Indonesia National Health Research and Development Unit Laboratory in Magelang.

To estimate prevalence of thyroid dysfunction via interpretation of thyroid hormone values, we applied threshold criteria. For TSH, we followed the 2017 American Thyroid Association (ATA) guidelines, which recommend, where possible, use of a population-specific reference range for TSH, since there are significant geographic and ethnic differences in TSH concentrations during pregnancy [23]. In the absence of relevant data from Indonesia and whilst wanting to take ethnic differences into account, we searched for proxy thresholds from another South East Asian country to apply in this case. We thus identified and applied a TSH reference range proposed from a study in n = 1947 healthy Thai adults with no thyroid disease or autoimmunity [24]. To adapt their thresholds for pregnant women, in the first trimester, in line with the 2017 ATA guidelines [23], we reduced the lower threshold by 0.4 mIU/L and the upper threshold by 0.5 mIU/L. The TSH thresholds applied in the present study are therefore 0.1–4.6 mIU/L in the first trimester, and 0.3–5.1 mIU/L in the second and third trimesters. For the interpretation of fT4, we applied the Human Reagent (Human Gesselschaft fur Biochemica and Diagnostica mbH, Max–Planck-Ring21 65205 Wiesbaden, Germany) kit-specific reference range for pregnant women of 0.8–2.2 ng/dL. Trimester-specific values were not available. To permit comparison with previous studies, ng/dL was converted to Standard International Units using the conversion coefficient 12.9 [25]. We used the following criteria to estimate the prevalence of thyroid dysfunction in the study population: subclinical hypothyroidism: elevated TSH, fT4 within range; overt hypothyroidism: elevated TSH and low fT4 or TSH >10 mU/L and fT4 within range; subclinical hyperthyroidism: low TSH and fT4 within range; overt hyperthyroidism: low TSH and elevated fT4; isolated hypothyroxinemia: TSH within range and low fT4 [23, 26, 27].

*Dietary iodine intake*. We estimated individual iodine intakes by asking participants about their dietary patterns, cooking methods, and the number of family members in the household. We used a food frequency questionnaire (FFQ) to assess the usual food intake and to examine the contribution of foods rich in native iodine. Using this information, we estimated the pattern of daily iodine intake from food. Iodine content of foods was estimated using results from Gunanti et al. [28]. To estimate the contribution of iodized salt, we used the duplicate portion method (described below) and the salt iodine concentration obtained by titration to estimate habitual iodine intakes from salt.

We collected household salt samples into a clean plastic container to measure the salt iodine concentration. All participants provided a sample, by duplicating the amount of salt that was used during cooking for one day. We divided the weight of the salt provided and divided it by the total number of persons in the household to obtain approximate individual intakes. Being an approximation only, the ages of household members were not taken into account for this calculation.

#### Statistical methods

Data management and statistical analyses were performed using IBM SPSS v21 (IBM Corp., Armonk, NY, USA). Data were tested for normality using the Kolmogorov Smirnov test. When data were not normally distributed, median, interquartile range (IQR) and 95% bootstrapped confidence intervals, based on 1000 bootstrapped samples, are reported, and Kruskall Walls and Mann-Whitney non-parametric tests were used to compare mean rank between trimester of pregnancy and between areas respectively.

We assessed iodine status based on the WHO/UNICEF/IGN criteria for pregnant women of a median UIC (MUIC): <150 μg/L indicating a risk of insufficient habitual iodine intakes; 150–499 μg/L an habitual sufficient iodine intake; and >500 μg/l habitual excessive iodine intakes. Logistic regression analysis was used to describe the relationship between trimester of pregnancy, risk of iodine deficiency, and thyroid dysfunction in both areas. First, a selection variable was used to identify the variable with the potential risk of thyroid dysfunction using the χ^2^ test, which was then analyzed via logistic regression to identify a prevalence ratio that was previously adjusted. The significance of all comparative tests was set at *p* <0.05.

## Results

Two hundred and forty-four pregnant women completed the study; 123 from area 1 and 121 from area 2. The average age of women was 28.5 ± 5.9 years, and average of parity 0.8 (range 1–3 children). As described previously, we assessed the nutrition status of pregnant women by measuring the MUAC. There was no difference between age and nutrition status of participants in each trimester, in both areas (Table 1).

**Table 1:** Participant characteristics, and urinary iodine concentration in spot urine samples with urinary iodine excretion in 24 urine samples.

		Trimester 1	n	Trimester 2	nn	Trimester 3	nn	All trimesters	nn
**Area 1**	**Age; y**	28.4 ± 5.2	37	27.4 ± 6.3	58	29.0 ± 7.0	28	28.1 ± 6.2	1123
	**Gestational Age; Mo**	10.6 ± 2.4^a^		20.5 ± 4.3^a^		33.4 ± 3.7^a^		20.4 ± 9.0	
	**MUAC; cm**	26.3 ± 3.7		26.1 ± 3.3		25.8 ± 2.6		26.1 ± 3.3	
	**Median UIC; μg/L**	253	37	211	58	179	28	222	123
	**IQR; μg/L**	145, 340		153, 296		108, 304		189, 276	
	**95% CI; μg/L**	198–351		155–262		134–294		186–253	
	**Mean UIE; μg/24 h**	224	37	232	58	197	28	221^d^	1123
	**SD**	99		87		70		88	
**Area 2**	**Age; y**	29.8 ± 5.3	36	28.5 ± 6.1	55	28.9 ± 5.5	29	28.9 ± 5.7	1121
	**Gestational Age; Mo**	10.7 ± 1.9^a^		20.8 ± 4.4^a^		32.9 ± 3.2^a^		20.8 ± 9.0	
	**MUAC; cm**	26.6 ± 4.0		26.4 ± 4.2		26.9 ± 2.8		26.6 ± 3.8	
	**Median UIC; μg/L**	279^a^	36	265^a^	55	193^a^	29	264	1121
	**IQR; μg/L**	106, 331		177, 310		132, 335		172, 284	
	**95% CI; μg/L**	251–363		210–332		134–270		231–286	
	**Mean UIE; μg/24 h**	240	36	252	55	232	29	244^d^	1121
	**SD**	77		105		82		92	
**Both Areas**	**Age; y**	29.1 ± 5.3	73	27.9 ± 6.2	113	28.9 ± 6.2	57	28.5 ± 5.9	2244
	**Gestational Age; Mo**	10.6 ± 2.1^a^		20.6 ± 4.2^a^		33.1± 3.4^a^		20.6 ± 9.0	
	**MUAC; cm**	26.4 ± 3.8		26.2 ± 3.8		26.3 ± 2.7		26.3 ± 3.6	
	**Median UIC; μg/L**	266^a^^,^^b^	73	237^a^^,^^b^	113	189^a^^,^^b^	57	246	2244
	**IQR; μg/L**	144, 317		189, 285		148, 295		198, 269	
	**95% CI; μg/L**	247–343		193–283		148–242		221–264	
	**Mean UIE; μg/24 h**	232	73	242	113	215	57	233	2244
	**SD**	89		97		77		90	

Unless otherwise indicated, values are mean ± SD or n (%).

IQR: interquartile range; SD: standard deviation; UIC: urinary iodine concentration from spot urine samples; UIE: urinary iodine excretion from 24 h urine samples. Y: year; mo: month; MUAC: mid-upper arm circumference. 95% CI were calculated using 1000 bootstrapped samples. Area 1: villages previously affected by severe iodine deficiency. Area 2: villages with no history of iodine deficiency. Median UIC thresholds in pregnant women are: <150 μg/L: risk of inadequate habitual iodine intakes; >500 μg/L: risk of excessive habitual iodine intakes.

^a^*p* value <0.05 (Kruskall-Walls) between trimester of pregnancy;

^b^*p* value <0.05 (Median test) between trimester of pregnancy;

^c^*p* value <0.05 (χ chi-square 3x2) between trimester of pregnancy;

^d^*p* value <0.05 (Mann Whitney test) between area 1 and area 2. Kolmogorov Smirnov test for UIE *p* >0.05.

### Urinary iodine concentration

Table 1 also describes the MUIC in spot urine samples and mean UIE. Mean UIE was higher in area 2 compared to area 1 (area 1: 221 ± 88 μg/L; area 2: 244 ± 92 μg/L; *p*<0.05 Mann Whitney test), however there was no difference in mean UIE across areas and in each trimester (*p*>0.05). MUIC decreased with trimester in both areas (*p* <0.05 between trimesters 1 and 3). Correspondingly, the proportion of pregnant women with MUIC <150 μg/L increased with trimester in both areas (*p* = 0.011) and the risk of UIC <150 μg/L increased with advancing trimester (OR = 2.59 (95% CI 1.19–5.67) at second trimester; OR = 3.85 (95% CI 1.64–9.02) at third trimester) in both areas.

### Thyroid function

Table 2 describes the median TSH and fT4 for pregnant women in both areas and from each trimester. Though values generally remained within our proxy thresholds (see methods), median TSH concentrations increased through gestation (*p* = 0.015 and 0.039 for area 1 and area 2, respectively; *p* = 0.001 for both areas together); whilst median fT4 in women decreased between trimesters in area 2, (*p* = 0.194 and 0.010 for area 1 and area 2, respectively; *p* = 0.003 for both areas together).

**Table 2:** Thyroid function and thyroid function disorders in the sample population across area 1 and area 2.

		Trimester 1	n	Trimester 2	n	Trimester 3	n	All trimesters	nn
**Area 1**	**Median TSH; mIU/L**	0.81^a^	37	1.39^a^	58	1.55^a^	28	1.30^a^	123
	**IQR; mIU/L**	0.64, 1.96		1.08, 2.05		0.79, 2.85		1.23, 2.04	
	**95% CI; mIU/L**	0.43–1.06		1.13–1.62		0.99–2.66		0.97–1.47	
	**Median fT4;**		37		58		28		123
	**ng/dL**	1.20;		1.13;		0.95;		1.12	
	**pmol/L**	15.4		14.5		12.2		14.4	
	**IQR;**								
	**ng/dL**	0.32, 0.55		0.35, 0.49		0.16, 0.63		0.38, 0.51	
	**pmol/L**	4.00, 7.10		4.55, 6.42		1.68, 8.00		4.84, 6.58	
	**95% CI;**								
	**ng/dL**	1.05–1.36		0.97–1.27		0.90–1.21		1.02–1.22	
	**pmol/L**	13.5–17.5		12.5–16.3		11.6–15.6		13.1–15.7	
	**Overt hypothyroid-ism**	0 (0.0)	37	0 (0.0)	58	0 (0.0)	28	0 (0.0)	1123
	**Subclinical hypothyroid-ism**	0 (0.0)		1 (1.7)		2 (7.1)		3 (2.4)	
	**Isolated hypothyroxin-emia**	1 (2.7)		4 (6.9)		0 (0.0)		5 (4.1)	
	**Overt hyperthyroid-ism**	0 (0.0)		0 (0.0)		0 (0.0)		0 (0.0)	
	**Subclinical hyperthyroid-ism**	4 (10.8)		7 (12.1)		2 (7.1)		13 (10.6)	
**Area 2**	**Median TSH; mIU/L**	0.94^a^	36	1.56^a^	55	1.70^a^	30	1.27^a^	1121
	**IQR; mIU/L**	0.75, 3.48		1.34, 2.78		0.83, 2.48		1.37, 2.37	
	**95% CI; mIU/L**	0.41–1.13		0.86–1.85		0.84–2.02		0.97–1.58	
	**Median fT4;**								
	**ng/dL**	1.30^b^		1.16		1.00^b^		1.12^b^	
	**pmol/L**	16.8^b^		14.9		12.9^b^		15.2^b^	
	**IQR;**								
	**ng/dL**	0.26, 0.55		0.31, 0.52		0.92, 1.11		0.35, 0.53	
	**pmol/L**	3.35, 7.06		3.93, 6.77		2.64, 6.35		4.45,6.93	
	**95% CI;**								
	**ng/dL**	1.24–1.49		1.07–1.29		0.18–0.50		1.11–1.27	
	**pmol/L**	15.9–19.4		13.8–16.6		11.9–14.9		14.2–16.5	
	**Overt hypothyroid-ism**	0 (0.0)	36	0 (0.0)	55	0 (0.0)	30	0 (0.0)	1121
	**Subclinical hypothyroid-ism**	0 (0.0)		3 (1.8)		1 (3.3)		2 (1.7)	
	**Isolated hypothyroxin-emia**	0 (0.0)		3 (5.5)		2 (6.7)		2 (1.7)	
	**Overt hyperthyroid-ism**	0 (0.0)		0 (0.0)		0 (0.0)		0 (0.0)	
	**Subclinical hyperthyroid-ism**	2 (5.6)		10 (18.2)		1 (3.3)		14 (11.5)	

Unless otherwise indicated, values are n (%). fT4: free thyroxine; IQR: interquartile range; TSH: thyroid-stimulating hormone. 95% CI were calculated using 1000 bootstrapped samples. Area 1: villages previously affected by severe iodine deficiency. Area 2: villages with no history of iodine deficiency.

^a^*p* value <0.05 (Kruskall-Walls test) between trimester of pregnancy;

^b^*p* value <0.05 (ANOVA) between trimester of pregnancy. Criteria applied to define thyroid function disorders: subclinical hypothyroidism: elevated TSH, fT4 within range; overt hypothyroidism: elevated TSH and low fT4 or TSH >10 mU/L and fT4 within range; subclinical hyperthyroidism: low TSH and fT4 within range; overt hyperthyroidism: low TSH and elevated fT4; isolated hypothyroxinemia: TSH within range and low fT4 [23, 26, 27].

Estimated prevalence rates of thyroid dysfunction in pregnant women is described in Table 2 by trimester, and in Table 3, according to UIC. No possible cases of overt thyroid disease were identified in this study. Subclinical hyperthyroidism was the most prevalent type of thyroid dysfunction (10.% and 11.5% in area 1 and area 2, respectively), though no difference was found between trimesters (*p* >0.05 across both areas). Taken together, the prevalence of isolated hypothyroxinemia and subclinical hypothyroidism increased with advancing trimesters of pregnancy (1.4%; 8.0%; 8.6%). The overall prevalence of thyroid dysfunction in the second trimester of pregnancy was higher than in the first and third trimesters in area 2 (trimester 1: 5.6%; trimester 2: 25.5%; trimester 3: 6.7%, *p* = 0.012; trimester 1–2 *p* = 0.015, trimester 1–3 *p* = 0.621, trimester 2–3 *p* = 0.035). In area 1, there was no difference in prevalence of thyroid dysfunction between trimesters (overall *p* = 0.913).

**Table 3:** Thyroid function disorders according to urinary iodine concentration in women across all trimesters.

	Thyroid dysfunction classification	n	UIC <150 μg/L	UIC 150–499 μg/L	UIC ≥500 μg/L	All	Risk^1^ (95% CI)
**Area 1**	**Overt hypothyroidism**	123	0 (0.0)	0 (0.0)	0 (0.0)	0 (0.0)	2.28 (0.54–9.66)
	**Subclinical hypothyroidism**		3 (7.7)	0 (0.0)	0 (0.0)	3 (2.4)	
	**Isolated hypothyroxinemia**		1 (2.6)	3 (4.0)	1 (11.1)	5 (4.1)	
	**Overt hyperthyroidism**	123	0 (0.0)	0 (0.0)	0 (0.0)	0 (0.0)	0.17 (0.02–1.40)
	**Subclinical hyperthyroidism**		1 (2.6)	10 (13.3)	2 (22.2)	13 (10.6)	
**Area 2**	**Overt hypothyroidism**	121	0 (0.0)	0 (0.0)	0 (0.0)	0 (0.0)	1.50 (0.27–8.21)
	**Subclinical hypothyroidism**		0 (0.0)	1 (1.2)	1 (6.7)	2 (1.7)	
	**Isolated hypothyroxinemia**		2 (7.7)	1 (1.2)	2 (13.3)	5 (4.1)	
	**Overt hyperthyroidism**	121	0 (0.0)	0 (0.0)	0 (0.0)	0 (0.0)	0
	**Subclinical hyperthyroidism**		2 (7.7)	9 (11.2)	3(20.0)	14 (11.5)	0.57 (0.12–2.75)
**Both areas**	**Overt hypothyroidism**	244	0 (0.0)	0 (0.0)	0 (0.0)	0(0.0)	1.92^1^ (0.65–5.62)
	**Subclinical hypothyroidism**		3 (4.6)	1 (0.6)	1 (4.2)	5 (2.0)	
	**Isolated hypothyroxinemia**		3 (4.6)	4 (2.6)	3 (12.5)	10 (4.1)	
	**Overt hyperthyroidism**	244	0 (0.0)	0 (0.0)	0 (0.0)	0 (0.0)	0
	**Subclinical hyperthyroidism**		3 (4.6)	18 (11.6)	5 (20.8)	26 (10.7)	0.33 (0.09–1.13)

Unless otherwise indicated, values are n (%).

^1^The risk was calculated using a 2x2 χ^2^ test between thyroid dysfunction and UIC (<150 μg/l and ≥150 μg/l) (0) as a reference category by logistic regression. Area 1: Area previously affected by severe iodine deficiency. Area 2: Area with no history of iodine deficiency. UIC thresholds in pregnant women are: <150 μg/L risk of inadequate habitual iodine intakes; >500 μg/L: risk of excessive habitual iodine intakes. Criteria applied to define thyroid function disorders: subclinical hypothyroidism: elevated TSH, fT4 within range; overt hypothyroidism: elevated TSH and low fT4 or TSH >10 mU/L and fT4 within range; subclinical hyperthyroidism: low TSH and fT4 within range; overt hyperthyroidism: low TSH and elevated fT4; isolated hypothyroxinemia: TSH within range and low fT4 [23, 26, 27].

Though not statistically significant, in both areas and in all trimesters of pregnancy, prevalence of hypothyroidism (subclinical, and hypothyroxinemia) was 1.9 times (95% CI 0.65–5.62) higher in pregnant women with UIC <150 μg/L (*p*>0.05), and of subclinical hyperthyroidism, 3.1 times (95% CI 0.8–10.5) higher in pregnant women with UIC >500 μg/L (*p*>0.05). These data are described in Table 3.

### Dietary iodine intake

The mean household salt iodine concentration in area 1 and area 2, was 40.7 ± 21.1 ppm and 40.3 ± 20.2 ppm, respectively (*p* = 0.91). The mean (SD) salt iodine content across both areas was 40.5 ± 20.6 ppm, which would correspond to an approximate UIC of 262 μg/l in pregnant women, assuming an average urine volume of 1.5 L during pregnancy and a 90% excretion in urine [29, 30]. This estimate is close to the median UIC of the overall sample in this study of 246 μg/l (Table 1).

In addition to iodized salt, women also ate foods rich in native iodine, including fresh and dried seafood, seaweed, salted fish and shrimp, although most of these foods are seldom consumed. Consumption of, and estimated iodine intakes provided by these foods are given in Table 4.

**Table 4:** Estimated consumption of foods rich in native iodine, and corresponding estimated dietary iodine intakes.

Source of iodine	Area 1	Area 2	Both areas
***Fresh seafood***			
**Mean ± SD (g/day)**	4.7 ± 10.4	1.8 ± 4.6	3.3 ± 8.3
**Estimated iodine contribution, μg (14.5 μg/100g)**	0.7 ± 1.5	0.3 ± 0.7	0.5 ± 1.21
**Rare /seldom (%)**	94.7	99.1	96.8
**Often /Usual (%)**	5.3	0.9	3.2
***Seaweed***			
**Mean ± SD (g/day)**	0.3 ± 1.5	0.2 ± 0.7	0.3 ± 1.2
**Estimated iodine contribution, μg (1.72μg/g)**	0.6 ± 2.6	0.3 ± 1.2	0.4 ± +2.1
**Rare /seldom (%)**	98.2	99.1	98.6
**Often / Usual (%)**	1.8	0.9	1.4
***Salted fish***			
**Mean ± SD (g/day)**	2.2 ± 4.4	1.4 ± 4.8	1.8 ± 4.6
**Estimated iodine contribution, μg (249.09 μg/100g)**	5.4 ± 11.0	3.5 ± 12.0	4.5 ± 11.5
**Rare /seldom (%)**	89.5^1^	97.1	93.1
**Often /Usual (%)**	10.5^1^	2.8	6.8
***Dried seafood***			
**Mean ± SD (g/day)**	9.2 ± 14.4	8.5 ± 11.1	8.9 ± 12.9
**Estimated iodine contribution, μg (88.45 μg/100g)**	8.1 ± 12.7	7.5 ± 9.8	7.9 ± 11.4
**Rare /seldom (%)**	79.8	87.6	83.6
**Often /Usual (%)**	20.1	12.4	16.4
***Shrimp***			
**Mean ± SD (g/day)**	1.1 ± 3.7	0.1 ± 0.5	0.6 ± 2.7
**Estimated iodine contribution, μg (82.4 μg/100g)**	0.9 ± 3.1	0.1 ± 0.4	0.5 ± 2.2
**Rare /seldom (%)**	99.1	100	99.5
**Often /Usual (%)**	0.9	0	0.4
***Iodized salt***			
**Consumption (FFQ) Mean ± SD (g/day)**	12.2 ± 6.1	10.5 ± 3.4	10.8 ± 4.6
**Content Mean ± SD (ppm)**	40.7 ± 21.1	40.3 ± 20.2	40.5 ± 20.6
**>30 ppm**	80.5	75.2	77.9
**<30 ppm**	19.5	24.8	22.1

^1^χ^2^ test. Rare / seldom: consumes food <2 times per week; Often/usual: consumes food >3 times per week. Estimations are made using food weight and a habitual patterns of food intake. Iodine content of foods was estimated using results from [28]. FFQ: Food Frequency Questionnaire.

## Discussion

With the introduction of iodized salt in Indonesia in 1994, Indonesia has progressively achieved national iodine sufficiency. An MUIC of 223 μg/L in SAC and 172 μg/L in pregnant women was recorded in the 2013 Household Survey [11]. In line with the 2013 survey, this cross-sectional study in two areas of Central Java, one with a history of severe iodine deficiency, indicates that habitual iodine intakes in pregnant women in both of these areas are within WHO/UNICEF/IGN recommendations [6] and are a reflection of the national status. Further, this study supports the necessity of adequately iodized salt as a vehicle to maintain the iodine status of pregnant women in locations both with longstanding iodine sufficiency and those with a history of iodine deficiency, where foods rich in native iodine are infrequently consumed.

Pregnancy is a risk period for maintaining optimal iodine intakes. Overall, maternal thyroid hormone production increases by 50% [31], leading to an increased iodine requirement for the duration of gestation [32]. However, specific changes in both maternal and fetal physiology during the different stages of pregnancy may influence the precise iodine requirements for each trimester. In the first trimester, maternal thyroid hormone synthesis must cover both maternal and fetal needs: the fetal thyroid gland is only formed at week 12 and not capable of iodine organification until about week 16–20 [33]. Moreover, the fetal thyroid does not reach autonomy until after birth and placental transfer of thyroid hormone must continue until the end of pregnancy. The increase in iodine required to sustain the increase in maternal thyroid hormone synthesis is underlined by an increase in maternal iodine loss, both via placental transfer and an increase in renal blood blow flow and glomerular filtration rate, which peaks during the second trimester [31]. The results of the present study show a tendency for MUIC to decrease with increasing trimester, leaving an increasing proportion of women at risk of inadequate intakes in their third trimester.

Subclinical hyperthyroidism was the most prevalent form of thyroid dysfunction observed in this study. It is defined as a decrease in TSH below the lower reference range, whilst thyroxine remains within range. Normal physiological changes during early pregnancy can induce suppression of TSH secretion by increasing human chorionic gonadotropin (hCG) which can cause transient gestational hyperthyroidism [31, 34]. Previous studies have found that subclinical hyperthyroidism during pregnancy has the lowest clinical impact on maternal health and perinatal outcomes [35].

In contrast, hypothyroidism during pregnancy can have adverse effects on the offspring [1]. This study showed a relatively low prevalence of subclinical hypothyroidism and isolated hypothyroxinemia. Subclinical hypothyroidism is common during pregnancy [36–39], and previous research has shown that hypothyroidism (overt, subclinical hypothyroidism, and hypothyroxinemia) may risk adverse effects on fetal neurodevelopment [40]. A study conducted in Chinese pregnant women living in areas with sufficient iodine intakes (median UIC 180 μg/L) showed a greater prevalence of subclinical hypothyroidism and hypothyroxinemia than overt hypothyriodism during the first half of gestation [41]. In the present study, an increase in the combined prevalence of subclinical hypothyroidism and hypothyroxinemia was found with advancing gestational period in both areas, irrespective of history of iodine deficiency. Increases in prevalence of hypothyroidism have been documented in response to increasing iodine intakes, such as after implementation of iodized salt [42–44]. Though such disturbances in thyroid function may be emphasized in susceptible individuals, particularly those exposed to chronic inadequate iodine intakes (such as the population in the previously iodine deficient area in this study), given that the salt iodization program in Central Java is now sufficiently longstanding to have allowed population adaptation, this is unlikely to be the case.

Universal salt iodization (USI) has been shown to be an effective fortification strategy to assure optimal population iodine intakes including in vulnerable groups [45]. Salt sampled in this study was titrated at 40 mg/kg and is adequately iodized compared to the Indonesian national guidelines for salt iodization [46]. Foods righ in native iodine were available, though infrequently consumed by participants in this study. This underlines the importance of adequately iodized salt to maintain optimal habitual intakes in this population.

The strengths of this study include the comparison of two districts in Central Java, Indonesia, with different historical iodine intake profiles. Further, we used both spot and 24 h urine samples to obtain a more accurate picture of iodine status. That said, 24 h samples are limited in that collection of a complete sample is difficult to control without the use of concomitant agents such as p-aminobenzoic acid [47] or estimation of creatinine, neither of which was not used here. Our study is also limited by a lack of assessment of thyroid autoimmunity in study participants. Increases in the prevalence of hypothyroidism may occur in response to the correction of iodine deficiency, and this may be associated with an increase in thyroid antibodies [48]. To examine the relationship between UIC and thyroid dysfunction, WHO/UNICEF/IGN thresholds were applied [6]. Since assessment of UIC from spot samples is representative of the iodine intake of that individual over the last 24 hours only, categorization applied to spot samples does not represent a strictly accurate picture of iodine stauts, therefore preventing firm conclusions about the association between iodine intake and thyroid function disorders. Finally, we applied proxy thresholds to categorise our data, to account for significant geographic and ethnic differences in TSH concentrations during pregnancy [23]. Since these are not local, population-specific and trimester-sepcific reference ranges, they allow for estimated prevalence rates only. That said, the overall general low prevalence of thyroid dysfunction in this population supports our conclusions.

## Conclusion

In conclusion, this study has shown that pregnant women in Central Java, in areas with longstanding iodine sufficiency and deficiency, habitually consume sufficient iodine through the consumption of iodized salt. However, women in their third trimester of gestation may be at risk of inadequate iodine intakes and a higher risk of thyroid dysfunction and we therefore emphasize the importance of iodine program monitoring and surveillance in this region.

## Supporting information

S1 Data (DTA)

## References

[1] Zimmermann MB. The effects of iodine deficiency in pregnancy and infancy. Paediatr Perinat Epidemiol. 2012 7;26(SUPPL. 1):108–17. 10.1111/j.1365-3016.2012.01275.x 22742605

[2] Gowachirapant S, Jaiswal N, Melse-Boonstra A, Galetti V, Stinca S, Mackenzie I, et al Effect of iodine supplementation in pregnant women on child neurodevelopment: a randomised, double-blind, placebo-controlled trial. Lancet Diabetes Endocrinol. 2017;5(11):853–63. 10.1016/S2213-8587(17)30332-7 29030199

[3] Hynes KL, Otahal P, Hay I, Burgess JR. Mild iodine deficiency during pregnancy is associated with reduced educational outcomes in the offspring: 9-year follow-up of the gestational iodine cohort. J Clin Endocrinol Metab. 2013 5;98(5):1954–62. 10.1210/jc.2012-4249 23633204

[4] Farebrother J, Naude CE, Nicol L, Sang Z, Yang Z, Jooste PL, et al Effects of iodized salt and iodine supplements on prenatal and postnatal growth: A systematic review. Adv Nutr. 2018;9(3). 10.1093/advances/nmy009 PMC5952947 29767700

[5] Ma FZ, Skeaff SA. Assessment of Population Iodine Status. In: Pearce EN, editor. Iodine Deficiency Disorders and Their Elimination. Boston, MA, USA: Springer International Publishing; 2017 p. 15–28.

[6] WHO, UNICEF, ICCIDD. Assessment of iodine deficiency disorders and monitoring their elimination. Programme Managers Guide. Geneva, Switzerland; 2007.

[7] Muhilal, Latief D, Kartono D, Permaesih D. Perubahan Prevalensi Gondok Dari Tahun 1980 Sampai Tahun 1998 (Change in Goitre Prevalence among School Children 1980–1998) [Internet]. Vol. 22, Penelitian Gizi dan Makanan. 1999 [cited 2020 Sep 12]. p. 1–4. Available from: https://media.neliti.com/media/publications/158085-ID-perubahan-prevalensi-gondok-dari-tahun-1.pdf

[8] Goh C. An Analysis of Combating Iodine Deficiency: Case Studies of China, Indonesia, and Madagascar [Internet]. Washington D.C., US; 2001 [cited 2020 Sep 12]. p. 1–27. (OED Working Paper Series). Available from: https://ieg.worldbankgroup.org/sites/default/files/Data/reports/iodine.pdf

[9] UNICEF. Review of National Legislation for Universal Salt Iodization: South Asia and East Asia and the Pacific. Bangkok; 2013.

[10] Ministry of Health, National Institute of Health Research and Development. Indonesia Basic Health Research (RISKESDAS) 2007 [Internet]. Jakarta, Indonesia; 2008 [cited 2020 Sep 17]. p. 64–5. Available from: http://labdata.litbang.kemkes.go.id/images/download/laporan/RKD/2007/Riskesdas_2007_English.zip

[11] Ministry of Health, National Institute of Health Research and Development. Indonesia Basic Health Research (RISKESDAS) 2013 [Internet]. Jakarta, Indonesia; 2013 [cited 2020 Sep 17]. p. 252–3. Available from: http://labdata.litbang.kemkes.go.id/ccount/click.php?id=10

[12] Kartono D, Moeljanto D. Total Goitre Rate (TGR), Ekskresi Iodium Urin (EIU) Dan Konsumsi Garam Beriodium Di Provinsi Jawa Tengah (Total Goiter Rate, Urinary Iodine Concentration and Consumptioon of Iodized Salt In Province of Central Java) [Internet]. Vol. 36, Buletin Panel Kesehatan. 2008 [cited 2020 Sep 12]. p. 91–8. Available from: https://media.neliti.com/media/publications/67189-ID-none.pdf

[13] Kartono D, Jahari AB, Soekirman S, Izwardy D. The Situation Of Urinary Iodine Concentration (UIC) Among School Age Children, Women at Reproductive Age and Pregnant Women in Indonesia: The Analysis Of Riskesdas 2013 [Internet]. Vol. 39, Gizi Indonesia, Journal of the Indonesian Nutrition Association. 2017 [cited 2020 Sep 12]. p. 49–58. Available from: https://www.persagi.org/ejournal/index.php/Gizi_Indon/article/view/207

[14] Dinas Kesehatan Kabupaten Magelang, (Magelang District Health Office). Laporan Surveilans GAKI (IDD Surveillance Report). Magelang; 2013.

[15] Dinas Kesehatan Kabupaten Magelang, (Magelang District Health Office). Laporan Surveilans GAKI (IDD Surveillance Report). Magelang; 2014.

[16] World Health Organization. Vitamin and Mineral Nutrition Information System (VMNIS). WHO Global Database of Iodine Deficiency: Indonesia [Internet]. 2006 [cited 2020 Sep 12]. Available from: https://www.who.int/vmnis/iodine/data/database/countries/idn_idd.pdf?ua=1n/

[17] Tim Yodisasi. Kabupaten Dati II Magelang. 1996. Penanggulangan Gangguan Akibat Kekurangan Iodium Kabupaten Magelang, Magelang. (Iodization team District Magelang. 1996. Combatting Iodine Deficiency Disorders Programme in Magelang District, Magelang). 1996.

[18] Sutrisna A, Knowles J, Basuni A, Menon R, Sugihantono A. Iodine Intake Estimation from the Consumption of Instant Noodles, Drinking Water and Household Salt in Indonesia Aang. Nutrients. 2018;10(3):324. 10.3390/nu10030324 PMC5872742 29517995

[19] Kesehatan Kabupaten Magelang.2004. Evalusi Laporan Penaggulangan GAKY di Derah Endemis Kabupaten Magelang. Magelang (Health Agency of Magelang. 2004. Evaluation Report Iodine Deficiency Combating Programme in Endemic area. Magelang). 2004.

[20] The American College of Obstetricians and Gynecologists. How your fetus grows during pregnancy [Internet]. ACOG. 2012 [cited 2020 Sep 17]. Available from: https://www.acog.org/patient-resources/faqs/pregnancy/how-your-fetus-grows-during-pregnancy

[21] Levy PS, Lemeshow S. Sampling of Populations. Fourth Groves RM, Kalton G, Rao JNK, Schwarz N, Skinner C, editors. Hoboken, New Jersey: John Wiley & Sons; 2008.

[22] Lwanga S., Lemeshow S. Sample size determination in health studies, A Practical Manual. Geneva: World Health Organization Press; 1991 1–77 p.

[23] Alexander EK, Pearce EN, Brent GA, Brown RS, Chen H, Dosiou C, et al 2017 Guidelines of the American Thyroid Association for the Diagnosis and Management of Thyroid Disease During Pregnancy and the Postpartum. Thyroid. 2017;27(3):315–89. 10.1089/thy.2016.0457 28056690

[24] Sriphrapradang C, Pavarangkoon S, Jongjaroenprasert W, Chailurkit LO, Ongphiphadhanakul B, Aekplakorn W. Reference ranges of serum TSH, FT4 and thyroid autoantibodies in the Thai population: The national health examination survey. Clin Endocrinol (Oxf). 2014;80(5):751–6. 10.1111/cen.12371 24266630

[25] LabCorp. SI Unit Conversion Table [Internet]. 2020 [cited 2020 Sep 7]. Available from: https://www.labcorp.com/resource/si-unit-conversion-table

[26] Stagnaro-Green A, Abalovich M, Alexander E, Azizi F, Mestman J, Negro R, et al Guidelines of the American Thyroid Association for the Diagnosis and Management of Thyroid Disease. Thyroid. 2011;21(10). 10.1089/thy.2011.0087 21787128 PMC3472679

[27] Carney LA, Quinlan JD, West JM. Thyroid Disease in Pregnancy. Am Fam Physician. 2014;89(4):273–8. 24695447

[28] Gunanti IR, Suhardjo S, Kusharto CM, Rimbawan R, Wirjatmadi B. Kandungan Iodium pada Beberapa Bahan Makanan di Daerah Pantai Endemik dan Non-endemik. Bul Penelit Sist Kesehat. 1999;3(1).

[29] Hurrell RF. Bioavailability of iodine. Eur J Clin Nutr. 1997 1;51 Suppl 1:S9–12. 9023472

[30] Lamberg BA. Iodine deficiency disorders and endemic goitre. Eur J Clin Nutr. 1993 1;47(1):1–8. 8422870

[31] Glinoer D. The Regulation of Thyroid Function in Pregnancy: Pathways of Endocrine Adaptation from Physiology. Endocr Rev. 2014;18(3):404–33. 10.1210/edrv.18.3.0300 9183570

[32] Mulyantoro DK. Perlukah Wanita Hamil Mendapat Suplementasi Iodium? (Iodine Supplementation for Pregnant Woman: Is it necessary?) [Internet]. Vol. 8, Media Gizi Mikro Indonesia. 2017 [cited 2020 Sep 12]. p. 137–50. Available from: https://ejournal2.litbang.kemkes.go.id/index.php/mgmi/article/view/523

[33] Connelly KJ, Boston BA, Pearce EN, Sesser D, Snyder D, Braverman LE, et al Congenital hypothyroidism caused by excess prenatal maternal iodine ingestion. J Pediatr. 2012;161(4):760–2. 10.1016/j.jpeds.2012.05.057 22841183 PMC4354797

[34] Moleti M, Trimarchi F, Vermiglio F. Thyroid Physiology in Pregnancy. Endocr Pract. 2014;20(6). 10.4158/EP13341.RA 24449667

[35] Dülek H, Vural F, Aka N, Zengin S. The Prevalance of Thyroid Dysfunction and Relationship with Perinatal Outcomes in Third Trimester Pregnants Who Apply to Tertiary Center. North Clin Istanbul. 2018;6(3):267–72. 10.14744/nci.2018.51422 PMC6790929 31650114

[36] Saraladevi R, Nirmala Kumari T, Shreen B, Usha Rani V. Prevalence of thyroid disorder in pregnancy and pregnancy outcome. Int Arch Integr Med. 2016;3(3):1–11.

[37] Teng W, Shan Z, Patil-Sisodia K, Cooper DS. Hypothyroidism in pregnancy. Lancet Diabetes Endocrinol. 2013;1(3):228–37. 10.1016/S2213-8587(13)70109-8 24622371

[38] Blatt AJ, Nakamoto JM, Kaufman HW. National Status of Testing for Hypothyroidism during Pregnancy and Postpartum. J Clin Endocrinol Metab. 2012;97(3):777–84. 10.1210/jc.2011-2038 22170721

[39] Sutandar M, Garcia-Bournissen F, Koren G. Hypothyroidism in Pregnancy. J Obstet Gynaecol Canada. 2007;29(4):354–6. 10.1016/S1701-2163(16)32437-9 17475129

[40] Maraka S, Ospina NMS, O’Keeffe DT, Espinosa De Ycaza AE, Gionfriddo MR, Erwin PJ, et al Subclinical Hypothyroidism in Pregnancy: A Systematic Review and Meta-Analysis. Thyroid. 2016;26(4):580–90. 10.1089/thy.2015.0418 26837268 PMC4827301

[41] Shan Z., Chen Y., Teng W., Yu X., Li C., Zhou W., et al A study for maternal thyroid hormone deficiency during the first half of pregnancy in China. Eur J Clin Invest. 2009;39(1):37–42. 10.1111/j.1365-2362.2008.02055.x 19087128

[42] Burgi H, M Kohler BM. Thyrotoxicosis incidence in Switzerland and benefit of improved iodine supply. Lancet. 1998;352(9133):1034 10.1016/S0140-6736(05)60076-1 9759751

[43] Pedersen IB, Laurberg P, Knudsen N, Jørgensen T, Perrild H, Ovesen L, et al Increase in Incidence of Hyperthyroidism Predominantly Occurs in Young People after Iodine Fortification of Salt in Denmark. J Clin Endocrinol Metab. 2006;91(10):3830–4. 10.1210/jc.2006-0652 16849408

[44] Laurberg P, Cerqueira C, Ovesen L, Rasmussen LB, Perrild H, Andersen S, et al Iodine intake as a determinant of thyroid disorders in populations. Best Pract Res Clin Endocrinol Metab. 2010;24(1):13–27. 10.1016/j.beem.2009.08.013 20172467

[45] Dold S, Zimmermann MB, Jukic T, Kusic Z, Jia Q, Sang Z, et al Universal salt iodization provides sufficient dietary iodine to achieve adequate iodine nutrition during the first 1000 days: A cross-sectional multicenter study. J Nutr. 2018;148(4):587–98. 10.1093/jn/nxy015 29659964

[46] Badan Standarisasi Nasional (National Standardization Agency of Indonesia). Garam konsumsi beryodium (Iodized Salt Standart): SNI 3556:2010 [Internet]. Badan Standarisasi Nasional. 2010 [cited 2020 Sep 12]. Available from: http://sispk.bsn.go.id/SNI/DetailSNI/8401

[47] Jakobsen J, Pedersen AN, Ovesen L. Para-aminobenzoic acid (PABA) used as a marker for completeness of 24 hour urine: Effects of age and dosage scheduling. Eur J Clin Nutr. 2003;57(1):138–42. 10.1038/sj.ejcn.1601505 12548308

[48] Taylor PN, Albrecht D, Scholz A, Gutierrez-Buey G, Lazarus JH, Dayan CM, et al Global epidemiology of hyperthyroidism and hypothyroidism. Nat Rev Endocrinol. 2018;14(5):301–16. 10.1038/nrendo.2018.18 29569622

